# Impact of Reformation of the Arthroplasty Register From Analogic to Digital-Based Data: Increased Completeness and Accuracy of Knee Revision Data

**DOI:** 10.1016/j.artd.2026.102125

**Published:** 2026-08-24

**Authors:** Jake von Hintze, Mika Niemeläinen, Keijo Mäkelä, Antti Eskelinen

**Affiliations:** a Coxa Hospital for Joint Replacement, Faculty of Medicine and Health Technologies, Tampere University, Tampere, Finland; b Department of Orthopaedics and Traumatology, Turku University Hospital, University of Turku, Turku, Finland

**Keywords:** Arthroplasty register, Revision knee arthroplasty, Revision completeness, Registry harmonization

## Abstract

**Background:**

Internationally, attempts have been made to harmonize arthroplasty registries to improve the comparison of outcomes. In Finland, a thorough reformation of the Finnish Arthroplasty register (FAR) was implemented because register data had become outdated. Both the data collection system and the registry contents, including the classification of revision indications, were reformed and data gathering became fully electronic in 2014. Here, we aim to assess how the reformation of the FAR data collection system has affected the completeness and accuracy of the reporting of the reasons for knee revision in Finland. We also describe the reform of FAR data in detail and compare the reasons for knee revision in different national joint arthroplasty registries.

**Methods:**

We identified 160 revision knee arthroplasties performed at our institution before reformation of the FAR (2013) and 189 revisions after the reformation (2016). Detailed information on these revisions was obtained from the hospital’s database and compared with information recorded in the FAR.

**Results:**

Registration completeness for the revision knee arthroplasties performed at our institution was 73% (117/160) in 2013 and 100% (189/189) in 2016. Moreover, after registry reform, the accuracy of registering periprosthetic joint infections also improved. Indeed, whereas only 65% of periprosthetic joint infections (31/48) were correctly recorded in the FAR in 2013, the corresponding figure was 98% (79/81) in 2016.

**Conclusions:**

Following the reformation of the FAR data collection system, both the accuracy and completeness of knee revision data improved. Additionally, the harmonization of arthroplasty registries internationally will enable a more coherent comparison of outcomes.

## Introduction

National joint arthroplasty registries have provided information on the outcomes of joint arthroplasty surgery since 1975 [1]. The Finnish Arthroplasty Register (FAR) was established in 1980, and the registration of joint arthroplasty surgery became obligatory in 1997 [2]. In recent years, the trend has been toward collaborating and harmonizing registries to achieve maximum benefits and to enable more detailed comparisons between registries [[3], [4], [5]].

**Figure 1:**
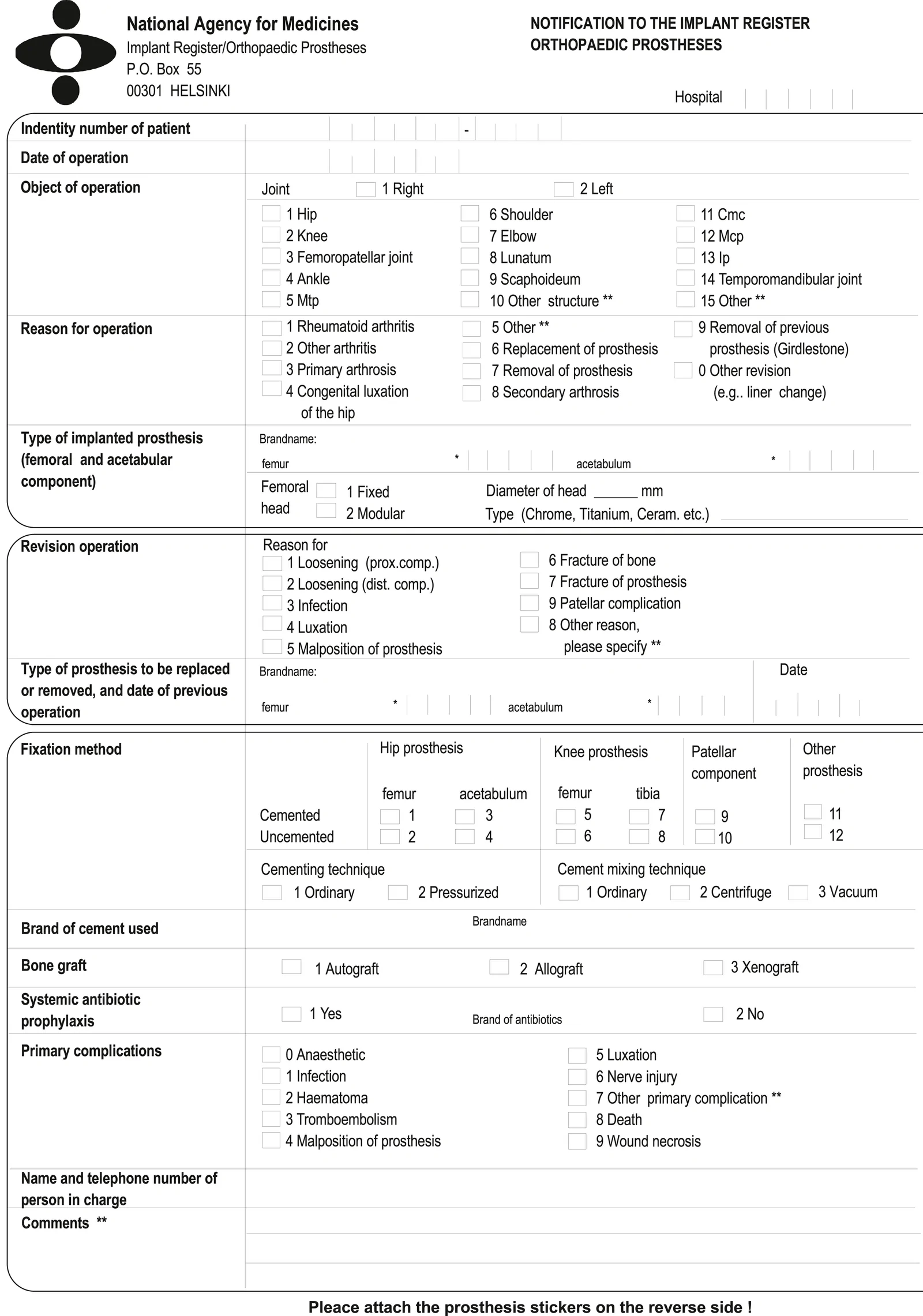
The notification form which was used before the year 2014.

**Figure 2:**
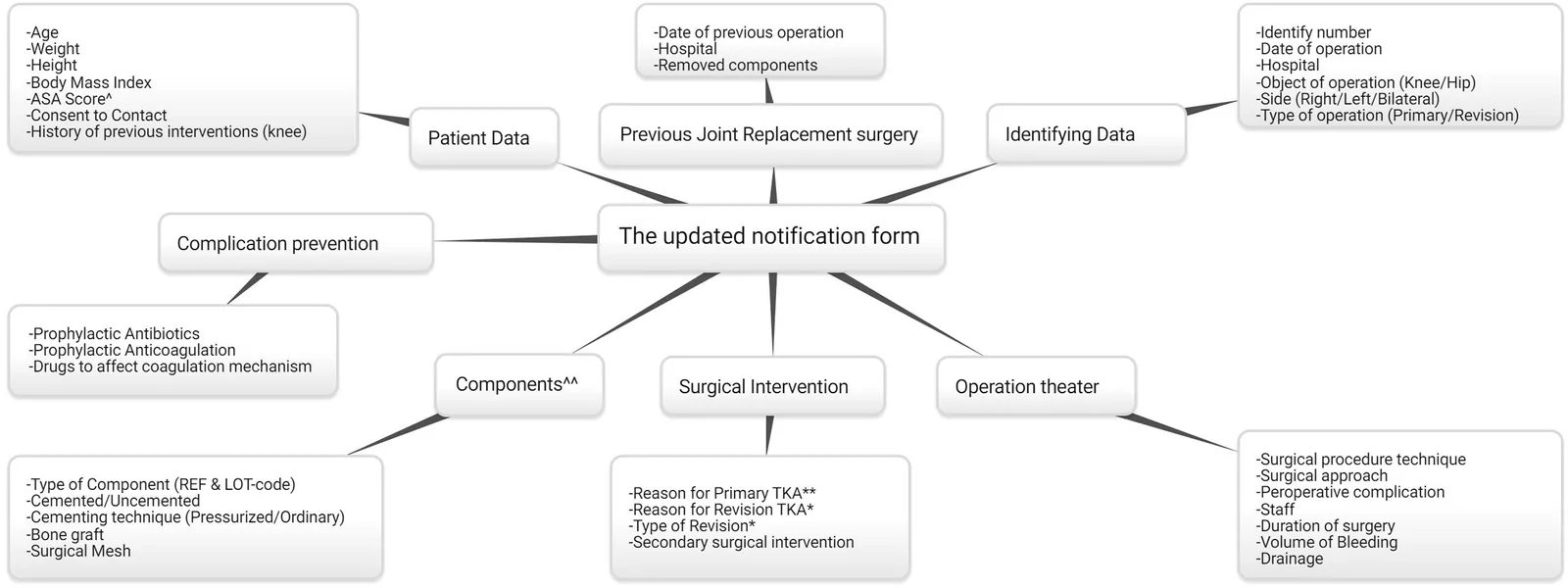
The updated electronic notification form for knee arthroplasty for the Finnish Arthroplasty Registry. ∗Only in revision TKA. ∗∗Only in primary TKA. ˆAmerican Society for Anesthesiologists score. ˆˆFemur, tibia, patella, and other augment components have own data.

Arthroplasty registries have played a significant role in tracking the outcomes of joint replacements [3,[6], [7], [8]]. In register research, the key outcome measure is implant survival. A revision operation is usually a clinically relevant, reproducible, and comparable end point [3]. In addition to implant survival, the quality of registry data is commonly assessed by measures such as completeness and accuracy [9]. Completeness refers to the extent to which all eligible procedures performed within the target population are captured by the registry, whereas accuracy describes how correctly the recorded data correspond to the original clinical information and procedures performed. High completeness and accuracy are essential for ensuring reliable outcome analyses and valid comparisons between registries. In a real-world setting, however, the data and variables registered in national registers may differ. For example, knee arthroplasty registries categorize the reasons for revision somewhat differently [10]. In Finland, the need for a thorough revision of the register data became obvious, as the reasons for knee revision were clearly outdated and the type of knee revision was often not recorded. Both the data collection system and the contents of the FAR were reformed between 2012 and 2014. Before the reform, the FAR annual reports were published in paper form and mostly included descriptive data. The notification form used prior to 2014 is presented in Figure 1. Since May 2014, data gathering has been all-electronic. The electronic notification form used today can be found on the Finnish Institute for Health and Welfare website (www.thl.fi/endo). Although the notification form is joint-specific (hip, knee, shoulder, elbow), it includes some patient data, such as body mass index and American Society of Anesthesiologists class. In addition, the form also includes more details about the surgery and exact information on the type of constraint in primary total knee arthroplasty (TKA) and the type of revision performed in revision TKA (eg, component-specific revisions, secondary patellar resurfacing, and one-stage or two-stage revision procedures for prosthetic joint infection [PJI]). Furthermore, the form has a wider variety of diagnoses regarding primary surgery and the reasons for revision. During surgery, the implants used are electronically scanned, and registration is based on the individual REF and LOT codes of the components. For the purposes of this study, we translated the current FAR knee notification form into English (Fig. 2). The notification form is completed in collaboration between the surgeon and the nurse in the operating theater.

These updates aimed to improve data quality and support future research.

The primary aim of this study was to assess whether the reformation of the FAR data collection system had subsequently affected the completeness and accuracy of the reporting of reasons for knee revision in Finland. To reach this goal, we investigated how knee revisions performed at the largest academic joint replacement hospital in Finland had been recorded in the FAR database prior to 2013 and after the reformation of the FAR in 2016. The second aim was to describe details of the reform of FAR data and compare the reasons for knee revision recorded in different national joint arthroplasty registries to evaluate the impact of the reform.

## Material and methods

The study hospital is an academic high-volume joint replacement center. From the electronic joint replacement register at our hospital, we identified all revision knee arthroplasties that were performed in 2 separate years: before the FAR reform (2013) and after the reform (2016). Revision was defined as a new operation during which at least one of the components was exchanged, removed, or added. Unicompartmental knee arthroplasty (UKA) to TKA conversions were included in the analysis. Second-stage procedures for PJI were excluded from the analysis.

In total, we identified 160 revision UKA/TKAs performed in 2013 and 189 in 2016 at our institution. The reason for each of the revisions was obtained from the hospital’s own register and cross-checked (by first author) from the medical records. There were no missing data, and no records were excluded due to incomplete data. From the FAR, we then obtained the reasons for all knee revisions performed at our institution during the study years (2013 and 2016). Data from our hospital’s local register were then compared to data from the FAR to find out, (a) the completeness of revision reporting (the proportion of revision procedures identified from hospital records that were captured by FAR) and the accuracy of revision reporting. Due to legal restrictions, individual revisions could not be matched between FAR and hospital records; therefore, reporting accuracy was inferred from the concordance of revision indication counts between the 2 data sources during the 2 study years; and (b) whether these measures improved after the reformation of the FAR data collection system and content.

To make an international comparison of knee registers in terms of the reasons for knee revision, the national registries were identified from the International Society of Arthroplasty Registries, the Network of Orthopaedic Registries of Europe website, and from the published literature [1,11,12]. We included those national registries that collect data on knee arthroplasty and have more than a 10-year follow-up period. In addition, the registers were discoverable in English, and information on the reasons for revision were available on the Internet. We excluded hospital- and regional-based registries from the analysis. We collected the reasons for the revision from the most current annual reports, online reports, and data collection forms. This work was supported by the competitive research funds of Pirkanmaa Hospital District, Tampere, Finland, representing governmental funding. Due to the retrospective nature of the present study, local ethical committee approval was not required, as the retrospective analysis only included data collected from the FAR and our institution’s database. In accordance with Finnish regulations, informed patient consent was not required, as the patients were not contacted. Data are presented as descriptive statistics with counts and percentages.

## Results

The reasons for revision in the 2 study years are shown in Table 1. The FAR contained the data of 117 revision knee arthroplasties in 2013 and 189 in 2016. Registration completeness in the FAR for the revision knee arthroplasty that were performed at our institution was 73% (117/160) in 2013 and 100% (189/189) in 2016. In both 2013 and in 2016, PJI was the most common reason for revision at our institution. The proportion of PJIs correctly registered in the FAR was 65% (31/48) in 2013 and 98% (79/81) in 2016. Although revisions due to patellar problems were the true reason for revision in 16.7% of knees at our institution in 2013, only 1.9% of revisions were classified as such in the old FAR database. In 2016, revisions for patellofemoral instability and revisions due to component malposition were registered in accordance with the institutional records. However, some discordance in the classification of revision indications persisted in 2016, particularly in the categories pain and other reasons. A total of 10 UKAs were revised at our institution in 2013. The reasons for revision were progression of knee osteoarthritis (OA) in 7 knees, insert dislocation in 2 knees, and pain in 1 knee. In 2016, a total of 13 UKAs were revised and the indication for revision was progression of knee OA in all cases.

**Table 1:** The reasons for revision overview from 2013 to 2016.

Our institution 2013 (TKA)	N (%)	FAR 2013 (TKA)	N (%)	Our institution 2016 (TKA)	N (%)	FAR 2016 (TKA)	N (%)
PJI	48 (32.0%)	PJI	31 (29.0%)	PJI	81 (46.0%)	PJI	79 (44.9%)
Patella problems^a^	25 (16.7%)	Patella problems^a^	2 (1.9%)	Loosening, tibia	6 (3.4%)	Loosening, tibia	6 (3.4%)
Loosening, tibia	7 (4.7%)	Loosening, tibia	4 (3.7%)	Loosening, femur	4 (2.3%)	Loosening, femur	1 (0.6%)
Loosening, femur	4 (2.7%)	Loosening, both	19 (17.8%)	Loosening, tibia + femur	2 (1.1%)	Loosening, tibia + femur	1 (0.6%)
Malposition	4 (2.7%)	Malposition	6 (5.6%)	Instability (TF)	46 (26.1%)	Instability (TF)	40 (22.7%)
Implant fracture, tibia	1 (0.7%)	Implant fracture	1 (0.9%)	Malposition, femur	3 (1.7%)	Malposition, femur	3 (1.7%)
Periprosthetic fracture, tibia	2 (1.3%)	Periprosthetic fracture	1 (0.9%)	Malposition, tibia	3 (1.7%)	Malposition, tibia	2 (1.1%)
Periprosthetic fracture, femur	1 (0.7%)					Malposition, patella	1 (0.6%)
		Luxation	4 (3.7%)	Insert wear	3 (1.7%)	Insert wear	2 (1.1%)
				Periprosthetic fracture, tibia	2 (1.1%)	Periprosthetic fracture, tibia	2 (1.1%)
Instability (TF)	34 (22.7%)	Other	39 (36.4%)	Periprosthetic fracture, femur	4 (2.3%)	Periprosthetic fracture, Femur	4 (2.3%)
Insert wear	10 (6.7%)			Instability (PF)	2 (1.1%)	Instability (PF)	2 (1.1%)
Stiffness	6 (4.0%)			Stiffness	7 (4.0%)	Stiffness	5 (2.8%)
Wound necrosis	1 (0.7%)			Pain	7 (4.0%)	Pain	17 (9.7%)
Iatrogenic MCL tear	1 (0.7%)						
Impingement	1 (0.7%)			Wound necrosis	1 (0.6%)	Other	11 (6.3%)
Iliotibial band syndrome	1 (0.7%)			Incorrect sizing	2 (1.1%)		
MCL tear	1 (0.7%)			Fascia tear	2 (1.1%)		
Hemarthrosis	2 (1.3%)			Impaired extensor mechanism	1 (0.6%)		
Pain	1 (0.7%)						
Total	150 (100%)		107 (65%)	Total	176 (100%)		176 (100%)

Our institution 2013 (UKA)	N	FAR 2013 (UKA)	N	Our institution 2016 (UKA)	N	FAR 2016 (UKA)	N
Progression of arthrosis	7	Loosening, both	7	Progression of arthrosis	13	Other	13
Insert luxation	2	Luxation	1				
Pain	1	Loosening, tibia	1				
		Other	1				
Total	10 (100%)	Total	10 (100%)	Total	13 (100%)	Total	13 (100%)

PF, patellofemoral; MCL, medial collateral ligament.

Registration completeness improved from 73% to 100%. The accuracy of the most common reason for revision (PJI) improved from 65% to 98%.

a Includes knee anterior pain and PF instability.

We identified 8 national registries that fulfilled the inclusion criteria for the analysis. The reported reasons for revision knee arthroplasty were collected from registries in Finland, Norway, Sweden, the Netherlands, Australia, New Zealand, Canada, and the National Joint Registry for England and Wales (NJR) [[13], [14], [15], [16], [17], [18], [19], [20]]. A summary of the reported reasons for revision are presented in Table 2. In the summary, both UKA and TKA revisions are combined because some registries do not specify these separately. Moreover, in some registries, certain reasons for revision are defined separately for each of the components. Among the registries, the number of reasons for revision variables varied from 9 (Sweden) to 32 (Australia).

**Table 2:** The summary of the reported reason for TKA/UKA revisions in the National arthroplasty registries.^a^

Reason for revision (TKA/UKA)	Finland	Sweden	Norway	UK	Australia	Canada	New Zealand	Netherlands
Infection	X	X	X	X	X	X	X	X
Instability	X	X	X	X	X	X	X	X
Loosening	X^c^	X	X^c^	X^c^	X	X	X^c^	X^c^
Periprosthetic fracture	X^c^	X	X	X^c^	X	X	X^d^	X
Progression of osteoarthritis/arthrosis		X	X	X	X	X	X	X
Insert wear	X	X		X	X^b^	X	X	X
Arthrofibrosis			X		X			X
Malalignment	X^c^^h^		X	X	X			X
Wound complications	X		X					
Implant fracture	X^b^			X	X^b^	X		
Insert luxation	X		X^k^		X			
Osteolysis	X^c^			X^d^	X	X		
Patella maltracking					X	X^g^		
Patella erosion					X			
Patellar dislocation/instability	X		X		X			X
Stiffness/contracture	X			X		X^f^	X^f^	
Pain	X		X	X	X	X^i^	X	
Other	X	X	X	X	X		X	X
Patella problems^e^		X						
Prosthesis dislocation/subluxation			X	X	X			
Osteonecrosis					X			
Defect polyethylene			X					
Patellar pain					X			X
Incorrect sizing					X			
Incorrect side					X			
Metal related pathology					X			
Synovitis					X			
Tumor					X			
Heterotopic bone					X			
Fracture osteosynthesis			X					
Extensor mechanism failure						X		
Remaining reasons^j^						X		
Revision after knee removal								X
Component dissociation				X				
Leg length discrepancy				X				
Missing			X					
Number of variables	24	8	18	20	31	13	12	14

^a^Registry information was obtained from the latest available registry versions (June 2026).

a The reason is defined for the tibia, femur, patella, and insert component.

b The reason is defined for the tibia, femur, and patella component.

c The reason is defined for the tibia and femur component.

d Includes all kinds of problems associated with the patella in patients that had their primaries inserted with or without a patellar button (excluding loosening and wear).

e Stiffness and arthrofibrosis are combined.

f Marked as patellar maltracking or instability.

g Marked as malposition.

h Pain and other complications are combined.

i Includes unspecified complications and diagnosis which are not generally related to knee replacement revision surgery (eg, cancer).

j Only for UKA.

## Discussion

The aim of the present study was to assess the success of the reformation of the data contained in the FAR. All-electronic data gathering may have contributed to the improved accuracy and reliability of the FAR data. Before the reformation, the number of revision operations recorded in the FAR and a single tertiary unit differed considerably. Furthermore, the proportion of “other reasons” given as reasons for revision has decreased markedly since the reform. There is, however, still room for improvement. For instance, although progression of osteoarthritis is one of the most common reasons for UKA revision, it is not specifically addressed in the FAR, even after the reform [14,17,20].

When the reporting system of the FAR was reformed in 2012-2014, an electronic registration system was introduced and traditional paper forms were abandoned. Interestingly, Puolakka et al. [2] had highlighted the need for this reform of the registration system already in the early 2000s. When surgery data details are recorded manually after the operation, the risk for human or transcription error may occur more often. Thus, an electronic registration system that is used in the operating room during the operation may offer more reliable data quality.

Electronic registration systems are also being used in the NJR, in Sweden, and in Canada [16,18,20]. Similarly, the Netherlands and Norwegian registers use both electronic and paper-based forms for data collection [14,19]. In Australia and New Zealand, details of the operations are still collected using traditional paper forms [15,17]. However, Australia has created the possibility for electronic registration, but the system is not in use at any site at present [17]. In terms of completeness, there is no radical difference between those registers that use paper forms and those that use electronic systems. Indeed, both the Australian and New Zealand registries show completeness rates of over 95% for primary procedures [9,21,22].

The Netherlands register reported excellent completeness for primary (99%) and revision (99%) knee procedures in 2024 [19]. Registers from Norway and Sweden have both reported high completeness rates for primary knee procedures, with completeness rates of 96% in Norway (2021-2023) and 98% in Sweden (2023). However, as was the case in the NJR, revision procedures are more complex. Thus, revision knee completeness was 92% in Norway (2021-2023) and 94% in Sweden (2023) [14,20].

Turppo et al. [23] validated information on primary joint arthroplasties between the FAR and the Finnish Hospital Discharge Register. They investigated 2820 primary arthroplasty events that had been recorded in the Finnish Hospital Discharge Register between 1980 and 2010. The vast majority (96%) of these events could also be found in the FAR. However, the findings of our study reveal that only 73% of the 161 revision knees performed at our institution in 2013 could be identified in the FAR data. To put it another way, 44 revision events were missing from the FAR database. By 2016, the completeness of this information had increased to 100%. Thus, all 189 revision knees performed at our institution in 2016 could also be found in the FAR database. Our cohort is, however, clearly smaller and comprised only revision knees. Still, the observed findings are consistent with potential benefits of the reform.

In addition, when different types of hospitals are compared, there is even more variation in the completeness of the registration [[24], [25], [26]]. According to Pedersen et al. [26] who reported from the Danish Hip Arthroplasty Registry, the completeness of the registration of hip revisions was highest (85.6%) when the surgery was performed at a high-volume hospital.

One of the explanations for the lack of completeness of registration could be a misunderstanding of the definition of revision. Debridement procedures without the exchange or removal of parts of the prosthesis can be problematic and not always considered a revision [25,27,28]. In their study, Lindgren et al. [29]reported that wound infections and debridement procedures without the exchange of parts of the prosthesis were well represented in nonregistered cases in the Swedish Hip Arthroplasty Register.

Our study revealed some discrepancies between the recording of the reasons for revision at our institution and the information obtained from the FAR database. In contrast to completeness, which reflects whether a revision procedure is captured by the registry, these discrepancies relate to the accuracy of the recorded revision indications. Afzal et al. [30] reported similar findings when they validated the reasons for revision data between a high-volume orthopaedic center and the NJR [18]. They determined 20.4% of the reporting of the reasons for revision TKA to the NJR had discrepancies. The most frequent discrepancies were in the reporting of progression of OA, malalignment, infection, and “other” [30]. In the present study, the discrepancy regarding PJI and tibiofemoral instability (TF) between our hospital and the FAR decreased after the reform of the register. TF instability was more typically reported as “other reason” or “malposition” before the reform of the register. In the current register, however, it has become the second most common reason for knee revision. In 2016, some discrepancies still remained, particularly in the reporting of TF instability and pain as reasons for revision. One possible explanation is that the underlying etiology of these conditions may not always be clearly identifiable at the time of surgery. This may lead to variation in how revision indications are categorized and reported. The proportion of revisions recorded as being performed for “other reasons” decreased from 34% in 2013 to 13% in 2016. Thus, the observed reduction in discrepancies is consistent with improved accuracy of the recorded reasons for knee revision following the register reform. Additionally, progression of OA (in UKAs) comprised more than half of “other reasons” in 2016. Interestingly, although the progression of OA is one of the most common reasons for UKA revision, it is not separately assessed as a reason for revision in the FAR, even after the reform [13,14,17,20].

In this context, Afzal et al. [30] highlighted that the reason for revision is recorded at the time of surgery. As microbiology reports take a few days to complete, the delay can result in an underestimation of the rate of revision for PJI. The possible updating of recorded registry data at the first scheduled follow-up visit could be one solution to this problem. In 2016, 2 out of the 81 (46%) knee revisions performed at our institution for PJI were incorrectly reported. It has been previously reported that national joint registries underestimate the rate of revisions for infection. Indeed, prior national arthroplasty registry studies have highlighted this trend [[27], [28], [29],^31^,^32^].

Niinimäki investigated the differences in the reasons for knee revision between arthroplasty registries. The author demonstrated that the reasons for revision knee arthroplasty were categorized differently among arthroplasty registries. Furthermore, it was also noted that pain, progression of OA, and stiffness/arthrofibrosis were variables that did not exist in all of the registries [10]. In the present study, we observed that revisions due to patella problems are recorded in several different ways. Of all the registries, the Swedish Registry can be considered the least detailed, as all patellar problems are included in 1 single reason for revision. In contrast, the Australian Registry has 6 different reasons for revision due to patellar problems. In total, the Australian Registry has 31 different reasons for knee revision. In Finland, the number of reasons for knee revision increased from 8 to 24 after the FAR reform. However, the FAR is still missing the systematic separate recording of UKA and TKA revisions, as is done in the Australian, New Zealand, Swedish, and Norwegian registries, and the NJR (Table 2).

We acknowledge that our study may have some limitations. First, we could not identify the reason for revision UKA/TKA from the FAR on a patient-by-patient basis. Current legislation on the secondary use of Health and Social Data complicated our efforts. Indeed, due to the current legislation in Finland, we were unable to track the discrepancies in reporting the reasons for revision between our institution and FAR. Moreover, we were also not able to determine the error rate for the reported reasons for revision. Second, some differences may have occurred due to a mismatch between our interpretation of the etiology of revision and the interpretation of the surgeons. The reasons for revision at our institution were examined from the local database and only 1 main reason for revision was recorded in the statistics. Moreover, detailed demographic data were not available, and therefore potential baseline differences between the 2013 and 2016 cohorts could not be assessed. Third, the number of revisions was too low before and after the reform of the registry to evaluate the revision data in more detail. In addition, as only two time points (2013 and 2016) were included, potential temporal trends in reporting and data quality beyond these years could not be assessed. However, the observed findings in this sample are consistent with improved accuracy and reliability of the recorded data. Moreover, the present study describes the reform process of a National Arthroplasty Registry and the changes in revision reporting observed following the reform.

## Conclusions

In conclusion, the findings of this study suggest that the reformation of a national joint arthroplasty register, including renewal of the registry content and implementation of an electronic registration system, may improve the accuracy and completeness of knee revision data. The possibility to easily update recorded registry data at the first scheduled follow-up visit could be one solution to improve accuracy, especially in cases of PJI. Additionally, registries need more harmonization so that the comparison of outcomes between registries would be more coherent.

## Funding

This work was supported by the competitive research funds of Pirkanmaa Hospital District, Tampere, Finland, representing governmental funding.

## Conflicts of interest

Mika Niemeläinen receives lecture fee from Johnson&Johnson. Antti Eskelinen receives lecture fees from Heraeus Medical GmbH and J&J Medtech; serves as an Advisory Board Member in Finnish Arthroplasty Register. All other authors declare no potential conflicts of interest.

For full disclosure statements refer to https://doi.org/10.1016/j.artd.2026.102125.

## CRediT authorship contribution statement

**Jake von Hintze:** Writing – original draft. **Mika Niemeläinen:** Writing – review & editing. **Keijo Mäkelä:** Writing – review & editing. **Antti Eskelinen:** Writing – review & editing, Supervision.

## Appendix A Supplementary data

Conflict of Interest Statement for Eskelinen

Conflict of Interest Statement for Niemeläinen

Conflict of Interest Statement for von Hintze

Conflict of Interest Statement for Mäkelä
